# Tomato SlCER1–1 catalyzes the synthesis of wax alkanes, increasing drought tolerance and fruit storability

**DOI:** 10.1093/hr/uhac004

**Published:** 2022-02-11

**Authors:** Hongqi Wu, Le Liu, Yaofeng Chen, Tianxiang Liu, Qinqin Jiang, Zhengyang Wei, Chunlian Li, Zhonghua Wang

**Affiliations:** State Key Laboratory of Crop Stress Biology for Arid Areas, College of Agronomy, Northwest A&F University, Yangling, 712100, China

## Abstract

Very-long-chain (VLC) alkanes are the main wax compounds of tomato fruits and leaves. ECERIFERUM1 (*CER1*) and ECERIFERUM3 (*CER3*) are the two key genes involved in VLC alkane biosynthesis in *Arabidopsis thaliana*. However, *CER1* and *CER3* homologs have not been investigated in tomato, and their exact biological functions remain unknown. We analyzed the wax profiles of tomato leaves and fruits at different growth stages and characterized the tomato *CER1* and *CER3* homologs. VLC alkanes were the predominant wax compounds in both leaves and fruits at all developmental stages. We identified five *CER1* homologs and two *CER3* homologs in tomato, which were designated *SlCER1–1* to *SlCER1–5* and *SlCER3–1* and *SlCER3–2*, respectively*.* The genes exhibited tissue- and organ-specific expression patterns and were induced by abiotic stresses. *SlCER1–1* was localized to the endoplasmic reticulum (ER), which is also the main site of wax biosynthesis. Silencing *SlCER1–1* in tomato significantly reduced the amounts of n-alkanes and branched alkanes, whereas its overexpression in *Arabidopsis* had the opposite effect. Under drought stress, both n-alkanes and branched alkanes increased significantly in wild-type but not *SlCER1–1* RNAi tomato plants. Furthermore, *SlCER1–1* silencing also increased the cuticular permeability of leaves and fruits. In conclusion, *SlCER1–1* is involved in wax alkane biosynthesis in tomato and plays an important role in drought tolerance and fruit storability.

## Introduction

The aerial surfaces of land plants are covered by a hydrophobic film known as the cuticle that protects against drought and other environmental stresses [1]. It consists of cutin, a polymer of C16 and C18 hydroxy and epoxy fatty acids, diacids and glycerol [2], and cuticular wax made of very-long-chain fatty acids (VLCFAs) and their derivatives, such as VLC alkanes, primary alcohols, secondary alcohols, ketones, aldehydes and triterpenoids [3, 4]. The composition and content of cuticular wax vary across species, tissues, developmental stages, and environmental conditions [4–6], underscoring its physiological relevance.

The wax biosynthesis pathway has been studied in *Arabidopsis thaliana* and involves de novo fatty acid synthesis, fatty acid elongation, and the formation of fatty acid derivatives [1], which are in turn completed by the alkane-forming and alcohol-forming pathways. In the alcohol-forming pathway, primary alcohols are produced directly through reduction by fatty acyl-CoA reductase (FAR); they then combine with acyl-CoAs to form wax esters in the presence of wax synthase/diacylglycerol acyltransferases (WSDs) [7–9]. The products of the alkane-forming pathway are aldehydes, alkanes, secondary alcohols, and ketones [1]. WAX2/YRE/CER3 reduces VLC acyl-CoAs to aldehydes [10–12], which are then decarbonylated to VLC alkanes by CER1 or CER1-like1 [13–16]. The VLC alkanes are further oxidized to secondary alcohols and ketones by MAH1 [17]. Studies show that CER1 and CER1-like1 physically interact with CER3 and form alkane-forming complexes to convert VLC acyl-CoAs to VLC alkanes of different chain lengths [15, 16]. Genes homologous to *CER1* and *CER3* have been identified in other species, including *BnCER1* in *Brassica napus* [18], *CsCER1* and *CsWAX2* in cucumber [19, 20], *ZmGL1* in maize [21], *WDA1*, *OsGL1s*, and *OsCER1* in rice [22–24], *TaCER1–1A* in wheat [25], *BdCER1–8* and *BdWAX2* in *Brachypodium distachyon* [26, 27], and *PpCER1–2* in *Poa pratensis* [28].

**Figure 1:**
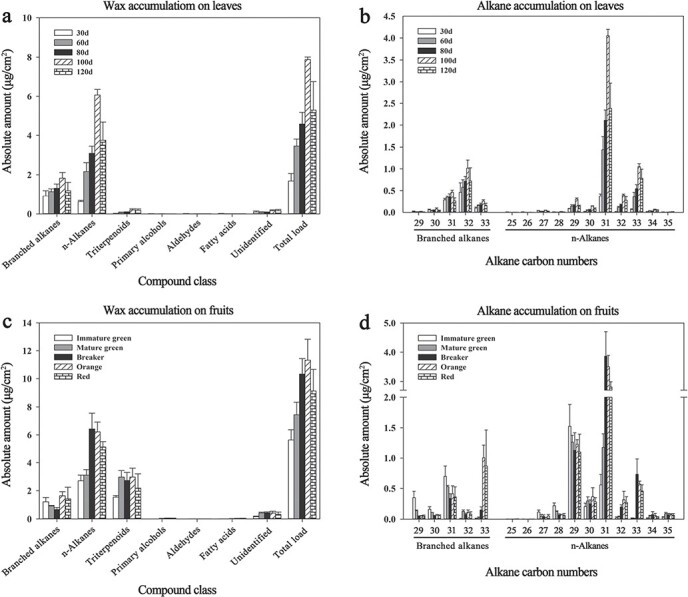
Developmental changes in cuticular wax accumulation on tomato leaves (**a** and **b**) and fruits (**c** and **d**). Numbers on the x-axis of (**b**) and (**d**) indicate carbon numbers of branched alkanes and n-alkanes. Amounts of individual wax constituents and total wax load are expressed as $ \mu $g/cm^2^ leaf or fruit surface area. Each value is the average of three biological replicates, and error bars indicate standard deviations.

The tomato fruit surface lacks stomata, and its cuticle has a relatively simple wax composition, making it an ideal model for studying the impact of cuticular wax on its barrier function [29–31]. Furthermore, tomato is also an important horticultural crop, and tomato mutants are valuable tools for genetic and genomic analyses [32]. Vogg et al. [29] showed that VLC aliphatic constituents in the intra-cuticular wax layer of tomato fruits play a more important role in preventing transpiration than does the epicuticular wax layer, but they are affected by the presence of triterpenoids. *SlCER6* encodes a β-ketoacyl-coenzyme A synthase involved in VLC fatty acid elongation in tomato [29, 30]. Fruits of the *slcer6* mutant exhibited a more significant increase in cuticular water permeability than the control, and this was caused mainly by a decrease in n-alkanes and a concomitant increase in triterpenoids [30]. Likewise, the complete absence of n-alkanes and aldehydes and the high content of triterpenoids and alkyl esters in the cuticular wax of tomato positional sterile (*ps*) mutant fruits significantly increases cuticular water permeability [31]. Thus, the proportion of n-alkanes and triterpenoids in the tomato fruit cuticle is most likely a determinant of cuticular water permeability.

Triterpenoid biosynthesis in the tomato fruit cuticle is driven by two oxidosqualene cyclases (OSC) [33]. The cuticular wax of tomato leaves is also dominated by VLC alkanes and triterpenoids, but their contents differ from those in tomato fruits [29], indicating that *CER1s*/*CER3s* and *OSCs* have tissue/organ-specific expression patterns. Indeed, Wang et al. [33] showed that both *OSCs* have distinct expression levels in the leaves and fruits of the tomato cultivar Micro Tom, corresponding to their respective triterpenoid contents. Nevertheless, studies to date have focused only on the cuticular wax composition of tomato leaves at specific growth stages [29, 34, 35], and the relationship between wax composition and cuticular barrier function has been elucidated only for tomato fruits [29–31]. Therefore, little is known about changes in leaf cuticular wax during development or the role of wax composition in leaf cuticular barrier function. To this end, we identified *CER1s* and *CER3s* in the tomato genome and functionally characterized a *CER1* candidate gene that is likely to be involved in VLC alkane synthesis and drought response.

## Results

### Wax accumulation patterns during leaf and fruit development in tomato

As shown in Fig. 1, the leaves and fruits of tomato had largely similar cuticular wax compositions. However, the cuticular wax composition of leaves was dominated by branched alkanes (21.94–53.71%) and n-alkanes (37.73–73.40%), whereas branched alkanes (6.42–21.54%), n-alkanes (40.67–61.38%), and triterpenoids (23.49–39.89%) were the major wax compounds in fruits. In addition, primary alcohols (0.02–0.59%, 0.03–0.38%), aldehydes (0.04–0.59%, 0.04–0.12%), and fatty acids (0.04–0.72%, 0.05–0.36%) were also detected in the leaves and fruits, and small amounts of triterpenoids (1.25–3.43%) were also present in the leaves (Fig. 1a and Table S1).

During leaf development, the total wax load increased steadily from 1.68 μg/cm^2^ on day 30 to 8.26 μg/cm^2^ on day 100 before decreasing to 5.29 μg/cm^2^ on day 120 (Fig. 1a and Table S1). The amounts of branched alkanes and n-alkanes also peaked at 100 days (Fig. 1a and Table S1), indicating that wax biosynthesis, especially n-alkane biosynthesis, is highest at this stage of development. The chain lengths of the n-alkanes ranged from C25 to C35, and C31 was predominant throughout development. The chain length of branched alkanes varied from C29 to C33, and the dominant chain length was C32 (Fig. 1b).

**Table 1:** General information on *SlCER* genes from tomato and characterized *CER1*/*CER3* genes from other species.

**Type**	**species**	**Gene symbol**	**Gene ID**	**CDS**	**Length (aa)**	**MW (kDa)**	**PI**	**Pfam matches**	
	Tomato	*SlCER1–1*	Solyc03g065250	1881	626	72.86	8.47	PF04116	PF12076
		*SlCER1–2*	Solyc01g088400	1887	628	73.20	8.45	PF04116	PF12076
		*SlCER1–3*	Solyc01g088430	1878	625	72.44	8.48	PF04116	PF12076
		*SlCER1–4*	Solyc12g100270	1863	620	71.51	8.53	PF04116	PF12076
		*SlCER1–5*	Solyc08g044260	1713	570	66.04	7.75	PF04116	PF12076
	*Arabidopsis*	*AtCER1*	At1g02205	1893	630	73.02	8.38	PF04116	PF12076
		*AtCER1-like1*	At1g02190	1884	627	72.09	7.44	PF04116	PF12076
	*Brassica napus*	*BnCER1–2*	KT795330	1935	644	74.63	8.02	PF04116	PF12076
	Cucumber	*CsCER1*	Csa024936	1857	618	71.38	8.79	PF04116	PF12076
CER1 homologs	Wheat	*TaCER1–1A*	MK214738	1860	605	68.88	8.25	PF04116	PF12076
	Rice	*Wda1*	OSJNBa0079L16.17	1866	621	71.22	8.24	PF04116	PF12076
		*OsCER1*	AK066386	1860	619	71.52	9.01	PF04116	PF12076
		*OsGL1–6*	AK068166	1908	635	71.64	8.64	PF04116	PF12076
	*Brachypodium distachyon*	*BdCER1–8*	Bradi3g28450	1865	621	71.04	8.90	PF04116	PF12076
	*Poa pratensis*	*PpCER1*	MH375602	1860	619	71.76	9.01	PF04116	PF12076
	Tomato	*SlCER3–1*	Solyc03g117800	1926	641	73.75	8.79	PF04116	PF12076
		*SlCER3–2*	Solyc07g006300	1893	630	72.55	8.87	PF04116	PF12076
	*Arabidopsis*	*AtCER3*	At5g57800	1899	632	72.29	8.78	PF04116	PF12076
	Cucumber	*CsWAX2*	Csa020530	1878	626	71.32	9.17	PF04116	PF12076
	Rice	*OsGL1–1*	AK060786	1860	619	69.65	9.37	PF04116	PF12076
		*OsGL1–2*	AK066569	1883	628	71.01	9.49	PF04116	PF12076
CER3 homologs		*OsGL1–3*	AK070469	1884	627	70.97	9.17	PF04116	PF12076

Note: CDS, coding sequence; bp, base pair; aa, amino acid; MW, molecular weight; kDa, kiloDalton; pI, isoelectric point.

During fruit development, the total wax load increased steadily from 5.64 μg/cm^2^ at the immature green stage to 11.32 μg/cm^2^ at the orange stage and then decreased to 9.13 μg/cm^2^ at the ripe red stage (Fig. 1c and Table S2). The n-alkane content increased most significantly during the transition from the immature green stage to the breaker stage, and it reached peak levels at the breaker stage. Triterpenoid levels increased significantly after the immature green stage (Fig. 1c and Table S2). Finally, branched alkanes decreased from the immature green stage to the breaker stage before increasing again until the red stage (Fig. 1c and Table S2). The carbon chain lengths of n-alkanes ranged from C25 to C35; C29 n-alkanes were predominant at the immature green stage, C29 and C31 n-alkanes at the immature green stage, and C31 n-alkanes between the breaker stage and the red stage. The chain length of branched alkanes varied from C29 to C33, and the C31 branched alkanes were predominant from the immature green stage to the breaker stage, whereas C33 branched alkanes dominated at the orange and red stages (Fig. 1d). Taken together, these results show that VLC alkanes were the predominant cuticular wax in tomato leaves and fruits throughout their development, indicating that *CER1s* and *CER3s* may play an important role in cuticle formation in tomato.

### Identification of *CER1* and *CER3* homologs in tomato

**Figure 2:**
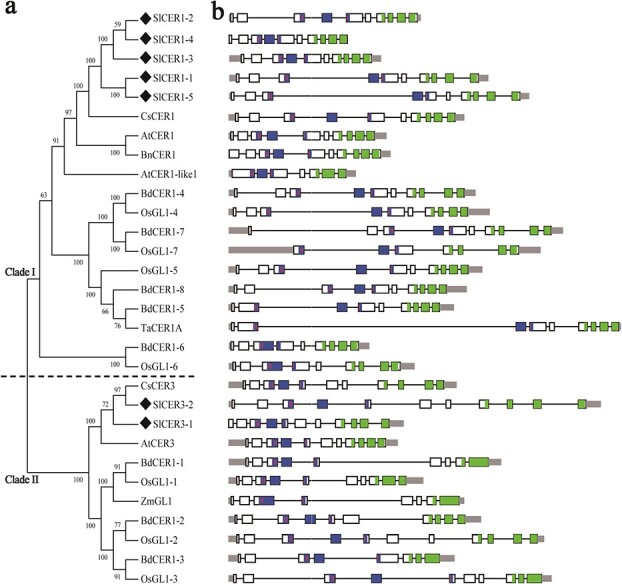
Phylogenetic tree and gene structure analysis of *CER1* and *CER3* genes from various species. (**a**) Phylogenetic tree. Black diamonds indicate candidate genes in tomato. *CER1* and *CER3* genes are present in Clade I and Clade II, respectively. At, *Arabidopsis thaliana*; Bn, *Brassica napus*; Cs, *Cucumis sativus*; Zm, *Zea mays*; Os, *Oryza sativa*; Bd, *Brachypodium distachyon*; Ta, *Triticum aestivum*; Sl, *Solanum lycopersicum*. (**b**) Gene structure. The black line indicates introns, gray rectangles indicate untranslated regions, and exons are represented as white, blue, and green rectangles (the FAH domains in blue contain the His-rich regions marked with red lines, and the WAX2 C-terminal domain is shown in green).

To identify tomato genes involved in VLC alkane formation, the CER1 and CER3 protein sequences from Arabidopsis were searched against the tomato genome database with BLASTP. Five *CER1* homologs (*SlCER1–1* to *SlCER1–5*) and two *CER3* homologs (*SlCER3–1* and *SlCER3–2*) were confirmed using the Pfam tool. Molecular characteristics of *SlCER* genes and characterized *CER1*/*CER3* genes are shown in Table 1. A total of 19 *CER1* and 11 *CER3* homologs from different species were used to build a phylogenetic tree, and two main clades were found: clade I, containing *CER1*-related proteins, and clade II, containing *CER3*-related proteins (Fig. 2a). *SlCER1–1* to *SlCER1–5* were classified into clade I and were more related to *AtCER1*, *AtCER1-like1*, *CsCER1*, and *BnCER1*. *SlCER3–1* and *SlCER3–2* were classified into clade II and were more related to *AtCER3* and *CsWAX2* (Fig. 2a).

Analysis of structural features revealed the presence of similar exon/intron structures and gene lengths in the *CER1* and *CER3* homologs (Fig. 2b). The FAH superfamily (blue rectangle, PF04116) and WAX2 C-terminal domain (green rectangle, PF12076) were the two key conserved domains in all *CER1* and *CER3* homologs (Table 1, Fig. 2b), and three His-rich motifs located in the FAH superfamily domain were also identified as a common feature (Fig. 2b). This analysis suggested that the *SlCERs* play an analogous role in wax alkane production in tomato.

### Expression patterns of *SlCER1s* and *SlCER3s* in different tissues or organs

**Figure 3:**
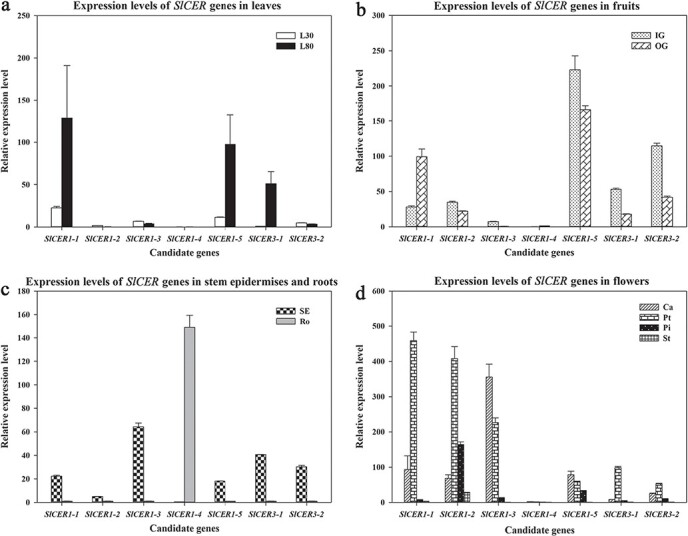
mRNA levels of *SlCER1s* and *SlCER3s* in different tissues and organs. (**a**) Expression patterns of *SlCER1s* and *SlCER3s* in leaves. L30 and L80 represent leaves from 30-day-old and 80-day-old plants. (**b**) Expression patterns of *SlCER1s* and *SlCER3s* in fruits. IG and Or represent the fruit epidermis at the immature green and orange stages. (**c**) Expression patterns of *SlCER1s* and *SlCER3s* in stems and roots. SE, stem epidermis; Ro, roots. (**d**) Expression analysis of *SlCER1s* and *SlCER3s* in floral tissues. Ca, calyxes; Pt, petals; Pi, pistils; St, stamens. Each value is the average of three biological replicates, and error bars indicate standard deviations.

Transcript levels of the *SlCER1s* and *SlCER3s* were measured in vegetative and reproductive organs. As shown in Fig. 3a, 3b and 3c, *SlCER1–1*, *SlCER1–5* and *SlCER3–1* were highly expressed in leaves, fruits, and stems, whereas *SlCER1–2* and *SlCER3–2* transcripts were detected mainly in fruits and stems. The expression of *SlCER1–3* was particularly high in stems. None of the *SlCER1s* and *SlCER3s* were detected in roots, with the exception of *SlCER1–4*, which was only expressed in this organ (Fig. 3c). Thus, all *SlCER* genes except *SlCER1–4* may be involved in VLC alkane biosynthesis in leaves, fruits, or stems, whereas *SlCER1–4* may have a root-specific function. Transcript levels of *SlCER1–1*, *SlCER1–5*, and *SlCER3–1* in leaves were significantly higher at 80 days than at 30 days (Fig. 3a). *SlCER1–1* showed higher expression in orange fruits compared with immature green fruits, whereas *SlCER1–2*, *SlCER1–5*, *SlCER3–1*, and *SlCER3–2* levels showed the opposite trend (Fig. 3b). Thus, the expression levels of these genes are under developmental control. Transcripts of several *SlCER1s* and *SlCER3s* were also detected in floral tissues like calyxes, petals, and pistils (Fig. 3d). Stamens showed low expression of *SlCER1–1* and higher levels of *SlCER1–2*, whereas *SlCER1–4* was not detected in any floral tissues. These results suggest that all genes except *SlCER1–4*, especially *SlCER1–2*, also contribute to VLC alkane biosynthesis in flowers. We searched the tomato functional genomics database to obtain information on the expression levels of all *SlCER* genes (Fig. S1). In general, the expression trends of *SlCER* genes in leaves and roots revealed in the transcriptome analysis were similar to those obtained by qRT-PCR.

To calculate the relative expression levels in each tissue, the expression values of *SlCER1–4* in pistils and other genes in roots were set to 1, respectively.

### *SlCER1s* and *SlCER3s* are induced by abiotic stresses

**Figure 4:**
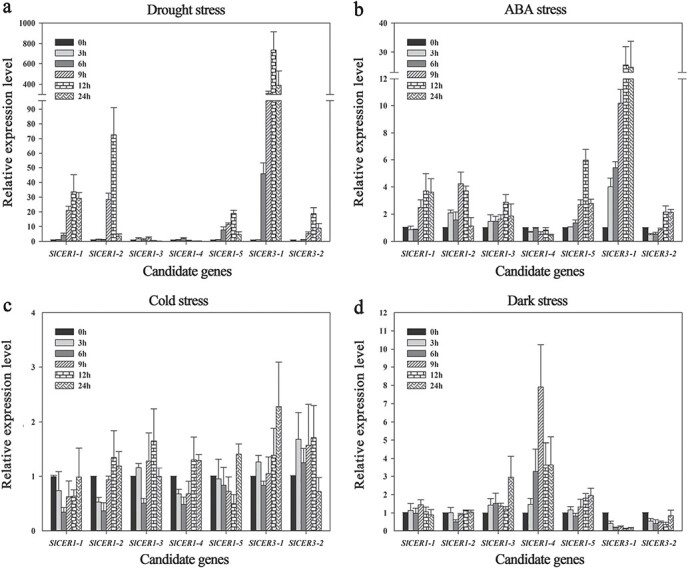
mRNA levels of *SlCER1s* and *SlCER3s* under (**a**) drought, (**b**) ABA, (**c**) cold, and (**d**) dark treatments. Each value is the average of three biological replicates, and error bars indicate standard deviations.

The promoter regions of *SlCER1s* and *SlCER3s* contain several elements related to drought, light, low-temperature, and ABA responses (Fig. S2). To confirm their involvement in responses to abiotic stresses, we analyzed the expression of *SlCER1s* and *SlCER3s* in one-month-old tomato plants exposed to various stress conditions. As shown in Fig. 4, all genes were induced by more than one stress. Drought conditions significantly upregulated *SlCER1–1*, *SlCER1–2*, *SlCER1–5*, *SlCER3–1*, and *SlCER3–2* expression by 34-, 73-, 19-, 736- and 19-fold, respectively, after 12 h. By contrast, the expression levels of *SlCER1–3* and *SlCER1–4* showed only a 2-fold increase at 9 h and 6 h, respectively (Fig. 4a). ABA treatment significantly upregulated *SlCER1–1*, *SlCER1–2*, *SlCER1–3*, *SlCER1–5*, *SlCER3–1* and *SlCER3–2* transcript levels by 4-, 4-, 3-, 6-, 26- and 2-fold, respectively, after 12 h (Fig. 4b), whereas *SlCER1–4* levels decreased with time (Fig. 4b). Cold exposure for 6 h or 12 h downregulated *SlCER1–1*, *SlCER1–2*, *SlCER1–4*, and *SlCER1–5*, which were restored to baseline levels thereafter. The expression levels of *SlCER1–3* and *SlCER3–2* decreased significantly after 6 h and 24 h of cold exposure, respectively, but showed a modest increase at other time points. By contrast, *SlCER3–1* expression increased gradually and was 2-fold higher at 24 h (Fig. 4c). Dark treatment resulted in a significant increase in *SlCER1–3*, *SlCER1–4*, and *SlCER1–5* expression, and *SlCER1–4* transcript level was upregulated by 8-fold after 9 h of exposure. However, *SlCER3–1* and *SlCER3–2* levels decreased over the 24-h treatment (Fig. 4d). Taken together, these results show that *SlCER1*s and *SlCER3*s are transcriptionally regulated in response to abiotic stresses.

### Subcellular localization of SlCER1–1

**Figure 5:**
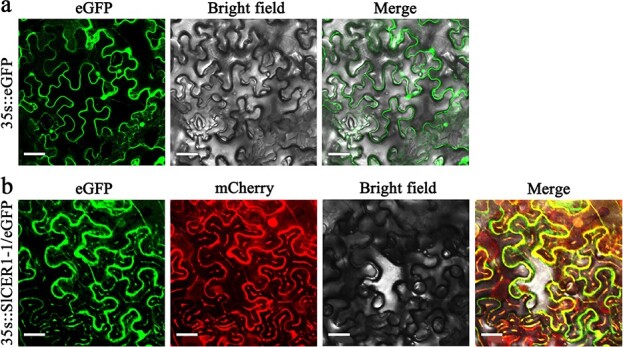
Subcellular localization of SlCER1–1 in *Nicotiana benthamiana*. (**a**) The eGFP signal of the empty vector. (**b**) SlCER1–1/eGFP (green) merged with the ER marker (red). The images from left to right show the eGFP signal of the empty vector or the SlCER1–1/eGFP fusion construct, the RFP signal of the ER marker mCherry-HDEL, and the bright-field image. The final image is a merge of the previous two or three images. Bars = 30 μm.

Given the high expression levels of *SlCER1–1* in different tissues and its significant induction by drought stress, we hypothesized that it may play a primary role in VLC alkane biosynthesis and drought tolerance. To examine the subcellular localization of SlCER1–1, we generated a construct harboring the *SlCER1–1* coding sequence (CDS) without the termination codon fused upstream of the enhanced green fluorescent protein (eGFP) gene driven by the CaMV 35S promoter. The construct was co-transformed with a red ER marker into *Nicotiana benthamiana* leaf epidermal cells. As expected, the green signal of the empty vector was found throughout whole cells (Fig. 5a), but the green signal of *SlCER1–1* co-localized with the red ER marker, and no green signal was observed in the nucleus (Fig. 5b). These results indicated that SlCER1–1 was located on the ER, where wax formation takes place.

### Overexpression of *SlCER1–1* enhances wax accumulation in *Arabidopsis*

**Figure 6:**
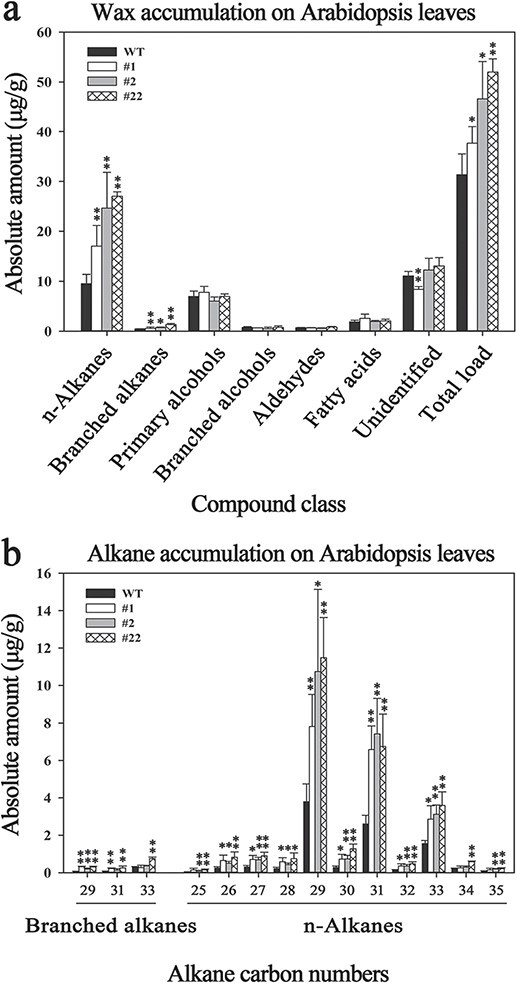
Overexpression of *SlCER1–1* in Arabidopsis. (**a**) Cuticular wax analysis of rosette leaves of wild-type and *SlCER1–1*-overexpressing *Arabidopsis* lines. (**b**) Chain length distributions and amounts of n-alkanes and branched alkanes on rosette leaves. The amounts of individual wax constituents and the total wax load are expressed as μg/g leaf fresh weight. Each value is the average of three to four biological replicates, and error bars indicate standard deviations. Asterisks indicate significant differences from the wild-type plants (*t*-test: *p < 0.05, **p < 0.01).

To further evaluate the biological function of *SlCER1–1*, we overexpressed it in *Arabidopsis*. Five T3 homozygous overexpression lines were obtained; lines 1, 2, and 22 showed the highest transcript levels in leaves (Fig. S3) and were therefore selected for the measurement of cuticular wax content by GC–MS and GC-FID. The total wax load in leaves was increased by 20.1%, 48.75%, and 65.84% in lines 1, 2, and 22, respectively, compared with that in wild-type plants (Fig. 6a, Table S3), and the n-alkanes and branched alkanes in particular were enhanced by 79.03%–184.34% and 21.91%–186.48%, respectively (Fig. 6a, Table S3). All chain lengths showed a significant increase, although the C29, C31, and C33 n-alkanes were predominant in all lines (Fig. 6b). By contrast, other wax compounds, including primary alcohols, branched alcohols, aldehydes, and fatty acids, showed no obvious changes (Fig. 6a, Table S3), indicating that *SlCER1–1* overexpression significantly induced wax accumulation by enhancing VLC alkane biosynthesis.

### Knocking down *SlCER1–1* reduced wax accumulation in tomato

**Figure 7:**
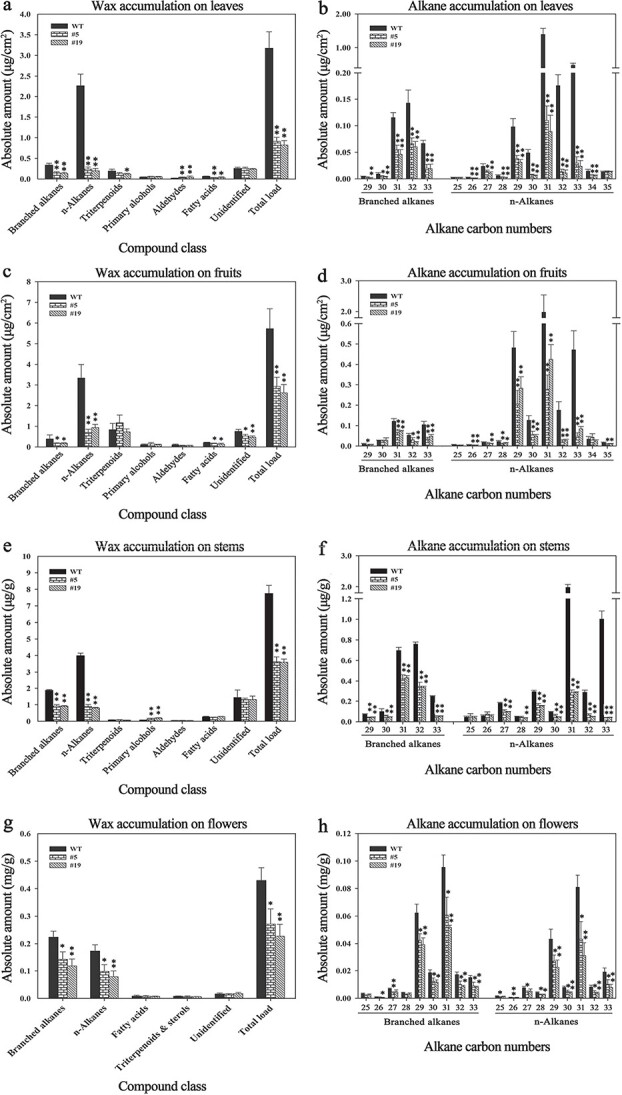
Cuticular wax analysis of wild-type and *SlCER1–1* RNAi plants. (**a**), (**c**), (**e**), and (**g**) Leaf wax composition and content at 60 days, fruits at red stage, stems at 40 days, and flowers without calyxes. (**b**), (**d**), (**f**), and (**h**) Chain length distribution and amounts of n-alkanes and branched alkanes in all organs. The amounts of individual wax constituents and the total wax load are expressed as μg/cm^2^ leaf or fruit surface area, μg/g stem fresh weight, or mg/g flower fresh weight. Data are means of three or four biological replicates, and error bars indicate standard deviations. Asterisks indicate significant differences from wild-type plants (*t*-test: ^*^p < 0.05, ^**^p < 0.01).

We also generated three *SlCER1–1* RNAi tomato lines (lines 2, 5 and 19). Lines 5 and 19 showed 87.05% and 74.84% decreases in transcript levels in leaves compared with the wild type, whereas line 2 showed only 29.47% downregulation (Fig. S4). Therefore, to determine the impact of *SlCER1–1* knockdown on wax composition and content, we analyzed the leaves, fruits, stems, and flowers of RNAi lines 5 and 19. The total cuticular wax content decreased by 70%, 50%, 53%, and 45%, respectively, in the leaves, fruits, stems, and flowers of the RNAi lines compared with the wild-type plants, and the greatest reductions were seen in the amounts of n-alkanes and branched alkanes (Fig. 7, Tables S4–S7). In addition, the fatty acid contents of the leaf and fruit waxes also decreased significantly after *SlCER1–1* knockdown (Fig. 7a, 7c, Tables S4, S5). Furthermore, the C29 to C33 branched alkanes and the C27 to C33 n-alkanes were predominantly decreased in all organs (Fig. 7b, 7d, 7f, 7h). Interestingly, the amounts of aldehydes in leaves and primary alcohols in stems were significantly increased in plants from line 5 (87.29%, 121.61%) and line 19 (109.23%, 162.47%) compared with the wild-type plants (Fig. 7a, 7e, Tables S4, S6), indicating that *SlCER1–1* knockdown reduces the conversion of aldehydes to alkanes and increases the content of primary alcohols. These findings strongly indicate that *SlCER1–1* is essential for VLC alkane biosynthesis in different parts of the tomato plant, especially leaves.

### Cuticular wax accumulation in the leaves of *SlCER1–1* RNAi plants during drought stress

**Figure 8:**
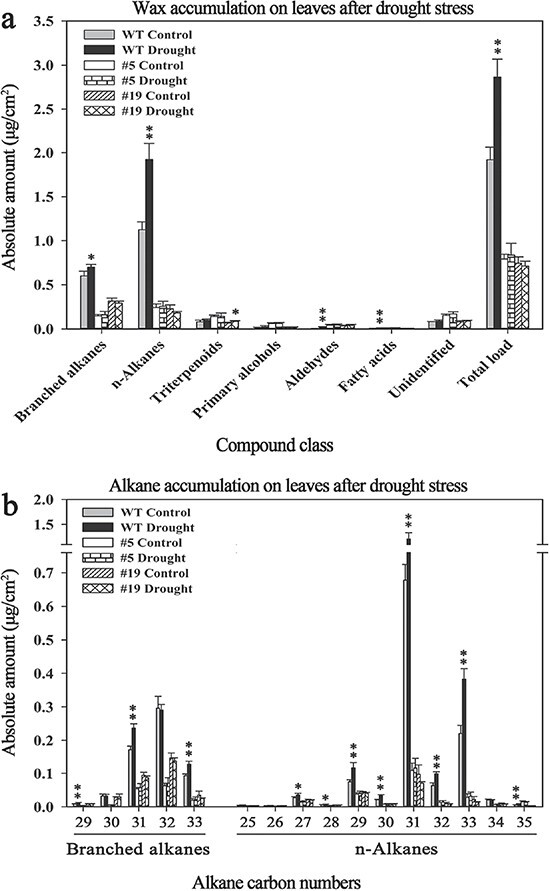
Cuticular wax content on leaves of wild-type and *SlCER1–1* RNAi tomato plants after drought stress. (**a**) Wax composition and content on leaves of wild-type and *SlCER1–1* RNAi tomato plants under control and drought conditions. (**b**) Chain length distributions and amounts of n-alkanes and branched alkanes. The total wax load and the amounts of individual components are expressed as μg/cm^2^ leaf surface area. The data are means of four biological replicates, and error bars indicate standard deviations. Asterisks indicate significant differences from plants grown under control conditions (*t*-test: ^*^p < 0.05, ^**^p < 0.01).

To assess the role of *SlCER1–1* during drought stress, we characterized the cuticular waxes isolated from the leaves of RNAi and wild-type tomato plants grown for 13 days under normal and drought conditions. Drought conditions strongly increased the wax load on leaves of wild-type plants, whereas no significant changes were detected in the RNAi lines (Fig. 8a, Table S8). In addition, the C27 to C33 n-alkanes and the C29, C31, and C33 branched alkanes were predominantly increased in drought-stressed wild-type plants (Fig. 8b), indicating a drought-responsive induction. Taken together, these results suggest that *SlCER1–1* is critical for wax accumulation and plays an important role in the drought stress response of tomato.

### Altered surface properties and drought tolerance in *SlCER1–1* RNAi plants

**Figure 9:**
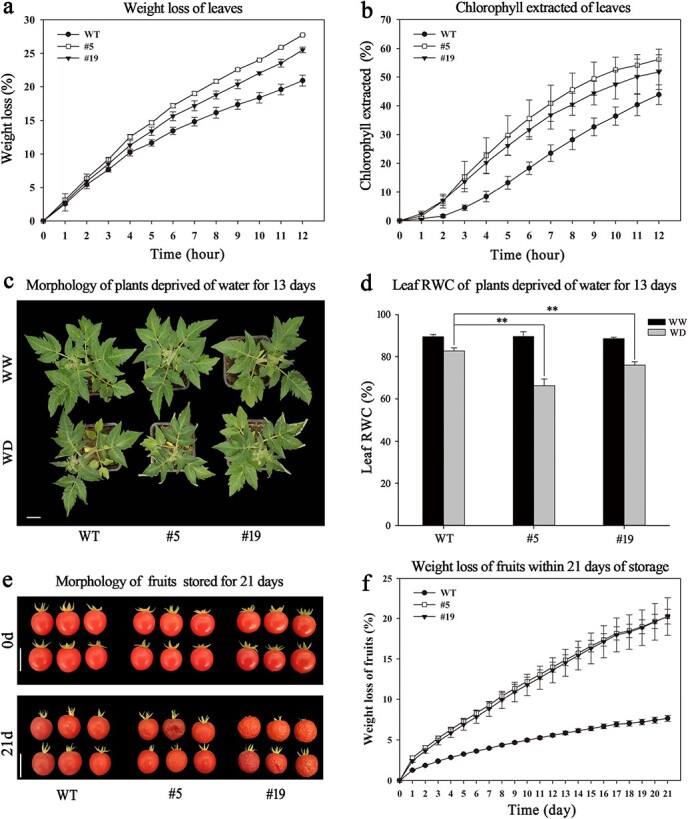
Surface permeability and drought response of *SlCER1–1* RNAi tomato plants. Water loss rates (**a**) and chlorophyll leaching rates (**b**) of isolated leaves from one-month-old wild-type and *SlCER1–1* RNAi tomato plants. (**c**) Drought resistance test of one-month-old wild-type and *SlCER1–1* RNAi tomato plants. These plants were well watered (WW) or deprived of water (WD) for 13 days. Bar = 3 cm. (**d**) Relative water content (RWC) of leaves from WT and *SlCER1–1* RNAi tomato plants in (**c**). (**e**) Morphology of tomato fruits stored for 0 and 21 days at room temperature. Bar = 3 cm. (**f**) Water loss rates of fruits from the WT and *SlCER1–1* RNAi tomato plants in (**e**). Data are the means of three biological replicates, and error bars indicate standard deviations. Asterisks indicate significant differences from water-deprived wild-type plants (*t*-test: *p* < 0.05, ^**^p < 0.01).

To determine whether reduced *SlCER1–1* expression and wax alkane production affect surface properties and drought tolerance, we measured water loss, chlorophyll leaching, and soil water deficit in the RNAi lines. Compared with the wild-type plants, the RNAi lines exhibited higher leaf water loss and faster leaf chlorophyll extraction at all time points (Fig. 9a and 9b). In addition, the leaves of the *SlCER1–1* RNAi plants exhibited “crinkled” edges after 13 days of water deprivation, indicating a stronger drought response compared with the wild-type plants (Fig. 9c). Consistent with this result, the relative water content (RWC) was significantly reduced in the drought-stressed RNAi lines compared with the wild-type plants grown under water-deprived conditions, whereas no significant difference was detected under well-watered conditions (Fig. 9d). Furthermore, the fruits of the RNAi lines were severely crinkled compared with the wild-type fruits after 21 days of storage at room temperature (Fig. 9e), and they also exhibited a higher water loss rate compared with the wild-type plants (Fig. 9f). Taken together, these findings show that *SlCER1–1* knockdown reduced wax alkane production in the cuticles of tomato leaves and fruits, which sensitized them to drought stress and reduced fruit storability.

## Discussion

VLC alkane biosynthesis in plant cuticles is catalyzed by the CER1 and CER3 enzymes. Previous studies have reported that the VLC alkanes and triterpenoids are the predominant wax components of tomato leaves and fruits [29–31, 34, 36, 37]. In addition, although two oxidosqualene cyclases have been identified that synthesize triterpenoids in the tomato fruit cuticle [33], little is known about the biosynthesis of VLC alkanes. In this study, we identified five *CER1* homologs (*SlCER1–1* to *SlCER1–5*) and two *CER3* homologs (*SlCER3–1* and *SlCER3–2*) in various tissues of the tomato plant, and we characterized *SlCER1–1* as the primary candidate gene involved in VLC alkane biosynthesis and drought stress response.

Analysis of the wax profiles of tomato leaves and fruits harvested at different stages confirmed the presence of n-alkanes throughout plant development. Given that n-alkanes are also the predominant cuticular wax component in different wild tomato species [38], their biosynthesis may play a key role in tomato cuticle formation. The SlCER1s and SlCER3s identified in this study possess the FAH superfamily domain, the WAX2 C-terminal domain, and three His-rich motifs that are indispensable for CER1 and CER1-like1 function in VLC alkane formation in *Arabidopsis* [15, 16], suggesting that they may have similar catalytic roles. *SlCER1–1*, *SlCER1–5*, and *SlCER3–1* were expressed at relatively high levels in leaves and fruits, which are the main sites of wax synthesis in tomato [29, 30, 36, 37]. In addition, the expression levels of *SlCER1–2* and *SlCER3–2* were high in fruits, and high levels of *SlCER1–2* were also detected in pistils and stamens, indicating their involvement in VLC alkane biosynthesis in these tissues. Smirnova et al. [39] found that mutations in the *SlCER6* gene caused floral organ fusion due to reduced VLC alkane production in flowers. Therefore, it is possible that *SlCER1–2* affects flower development via VLC alkane biosynthesis, a topic that is worthy of further investigation. *SlCER1–4* was preferentially expressed in roots, implying that it may be involved in wax alkane accumulation in roots or have other yet unknown functions.

Wax accumulation under water deficit has been correlated with the upregulation of *CER1*, *CER2*, *CER3*, *CER4*, *CER10*, and *WSD1* in grape berries [5] and *WSDs* in Arabidopsis [9]. *SlCER1s* and *SlCER3s* also showed a drought-dependent expression pattern in leaves and may therefore increase VLC alkane accumulation as part of the drought response. ABA is an important regulator of plant responses to drought and other abiotic stresses [40]. *MYB94* and *MYB96* are ABA-inducible transcription factors in *Arabidopsis* that bind directly to conserved motifs in the promoters of several wax biosynthesis genes in response to drought [41, 42]. Wax biosynthesis in tomato leaves is also regulated by ABA [34], and ABA treatment significantly upregulated all *SlCER1*s and *SlCER3*s except *SlCER1–4*. In addition, MYB binding consensus sequences were also present in the promoter regions of *SlCER* genes (Fig. S2). Therefore, we hypothesized that the *SlCER* genes may improve drought tolerance by enhancing VLC alkane accumulation following ABA-mediated stimulation, and they may also be regulated by *MYB94* or *MYB96* homologs.

Cold treatment significantly reduces the amounts of VLC alkanes in *Thellungiella salsuginea* and *Arabidopsis*, mainly by downregulating the expression of *CER1* and *CER3* [6, 43]. Cold stress also markedly downregulated *SlCER1–1*, *SlCER1–2, SlCER1–4*, and *SlCER1–5* in our study, suggesting that these genes are likely to play an important role in regulating the wax production of tomato under cold stress. The deposition of cuticular wax in *Arabidopsis* is diurnally regulated by the transcription factors SPL9 and DEWAX, which rhythmically regulate *CER1* expression [44, 45]. The expression levels of *SlCER1–3*, *SlCER1–4*, and *SlCER1–5* were upregulated in tomato in the dark, indicating that *SlCER1s* may be involved in similar diurnal regulation of wax accumulation in tomato. Furthermore, the 8-fold increase in *SlCER1–4* after 9 h in the dark suggested that nocturnal wax deposition in tomato is driven by upregulation of *SlCER1–4*. This dynamic of wax biosynthesis in tomato requires further investigation.

Several studies have shown that the enzymes involved in the synthesis of VLC alkanes, primary alcohols, alkenes, wax esters, and ketones are localized to the ER [9, 17, 19, 25, 34, 46]. The predominant localization of *SlCER1–1* in the ER confirmed that it may be the primary location of wax production in tomato as well. Furthermore, overexpression of *SlCER1–1* in *Arabidopsis* significantly increased the amounts of n-alkanes and branched alkanes in leaves, whereas its knockdown in tomato greatly reduced these wax components in leaves, fruits, stems, and flowers. Interestingly, *SlCER1–1* did not show any substrate chain length preference for the synthesis of C25 to C35 alkanes, and it also used branched substrates to form branched alkanes in both tomato and *Arabidopsis*. In addition, VLC alkanes were not completely absent in the different organs of *SlCER1–1* RNAi plants, indicating functional redundancy among the *SlCER1s*.

Given the considerable sequence homology between the different *SlCERs*, we also analyzed the expression levels of other homologs in the leaves of the *SlCER1–1* RNAi plant lines (Fig. S5). Although *SlCER1–2* and *SlCER1–3* were only slightly downregulated, the expression of *SlCER1–1* was strongly decreased. By contrast, the other genes were significantly upregulated. This result clearly indicated that the phenotypes observed in the RNAi lines were the result of *SlCER1–1* silencing. We identified a reduced VLC alkane mutant designated *slcer1-1* that did not exhibit floral organ fusion like *slcer6* [39], probably because the loss of *SlCER1–1* had a weaker inhibitory effect on VLC alkane synthesis than the loss of *SlCER6.* For instance, n-alkanes with chain length C31 or greater and branched alkanes with chain length C32 or greater are completely absent from the *slcer6* mutant anthers [39], whereas only a partial reduction in these compounds was observed in *slcer1–1* flowers. Interestingly, the significant reduction in n-alkanes with a concomitant increase in triterpenoids strongly enhances cuticular water permeability in the *slcer6* mutant fruits [30], raising the possibility that reduced VLC alkane content in the cuticles affects the surface permeability of *slcer1–1* mutant tomato plants as well.

Drought stress significantly increased the amount of VLC alkanes and marginally affected other wax components in wild-type tomato, consistent with observations in *Arabidopsis*, saltwater cress, and *Populus euphratica* [47–49]. However, no change was seen in the cuticular wax composition of water-deprived *slcer1–1* mutants, suggesting that the biosynthesis of VLC alkanes is inhibited in the absence of *SlCER1–1* under drought stress. Consistent with this notion, the leaves of *slcer1–1* mutants showed higher water loss, faster chlorophyll leaching, and lower RWC, which sensitized the plants to drought stress. Loss of function of other *CER1-like* genes such as *AtCER1* [14], *OsGL1s* [23], and *CsCER1* [20], resulted in similar phenotypes in *Arabidopsis*, rice, and cucumber, respectively. A recent study showed that VLC alkanes on the peels of apples play an important role in their storability [50]. Likewise, the decrease in VLC alkane accumulation in *slcer1–1* fruits enhanced water loss, resulting in significant crinkling. Thus, increasing the amount of VLC alkanes in tomato through traditional breeding or gene editing can improve drought tolerance and fruit storability.

In conclusion, VLC alkanes are the predominant compounds in cuticular wax of tomato leaves and fruits, and *SlCER1s* and *SlCER3s* are genes involved in their biosynthesis. Enhancing VLC alkane production in tomato leaves and fruits may improve drought tolerance and fruit storability. Furthermore, the *slcer1–1* mutant is a promising model for studying the functions of other *CER1* homologs in tomato or other species.

## Materials and methods

### Plant materials and growth conditions

The “Micro Tom” cultivar was used for all experiments. All tomato plants were grown in a greenhouse under a 14-h light/10-h dark cycle with temperatures ranging from 18 to 30°C during the day-to-night transitions. Sterilized seeds of Columbia (Col-0) *Arabidopsis* were planted on half-strength Murashige and Skoog (MS) medium containing 1% sucrose and incubated at 4°C for three days in the dark. They were then moved to growth chambers maintained at 22°C with a 16-h light/8-h dark cycle. After two weeks, *Arabidopsis* seedlings were transferred to soil and grown under the same conditions as above.

### Identification of *CER1* and *CER3* homologs in tomato

The tomato genome (http://solgenomics.net/) was queried with AtCER1 (NP_001184890) and AtCER3 (NP_200588), and the conserved domains of all putative non-redundant homologous protein sequences were confirmed using Pfam (http://pfam.xfam.org/search). Putative homologous protein sequences harboring the fatty acid hydroxylase (FAH) superfamily (accession no. PF04116) and WAX2 C-terminal (accession no. PF12076) domains were used as candidate *SlCER1*s and *SlCER3*s for further analysis.

### Sequence alignment, phylogenetic analysis and prediction of promoter cis-acting elements

The sequences of *CER1* and *CER3* homologs from other species were retrieved from the NCBI database (http://www.ncbi.nlm.nih.gov). Their exon-intron structures were examined using the online Gene Structure Display Server 2.0 (GSDS, http://gsds.gao-lab.org). Their molecular weights and theoretical isoelectric points were predicted using the Expasy ProtParam tool (http://web.expasy.org/protparam/). A phylogenetic tree was constructed using MEGA5 software based on the neighbor-joining method with the option of p-distance, pairwise deletion, and 1000 bootstrap replicates [51]. About 2000 bp of sequence upstream of the start codon of each *SlCER1* and *SlCER3* was submitted to PlantCARE (http://bioinformatics.psb.ugent.be/webtools/plantcare/html/) to predict the promoter cis-acting elements.

### Quantitative real-time PCR

After germination, a variety of tissues were collected to analyze the tissue expression patterns of the *SlCER1s* and *SlCER3s*: the top leaves of the fourth and fifth branches of 30-day-old and 80-day-old plants; the epidermis with some underlying cell layers from immature green and orange fruits; pistils, stamens, petals, and calyxes of flowers; and stem epidermis and roots from two-month-old plants. To analyze expression patterns of the *SlCER1s* and *SlCER3s* under abiotic stresses, one-month-old tomato seedlings were exposed to drought, ABA, cold or dark. For drought treatment, plants were washed out from pots, surface water was gently wicked from the roots using napkins, and the plants were transferred to dry filter papers. For ABA treatment, the plants were first irrigated with 100 ml ddH_2_O and then sprayed evenly with 100 μM ABA. For cold and dark treatments, the plants were irrigated with 100 ml ddH_2_O and then transferred to 4°C or dark conditions, respectively. Control plants were supplied with the same volume of ddH_2_O and grown under normal conditions. Top leaves were sampled from the fourth and fifth branches at 0, 3, 6, 9, 12, and 24 h of each treatment. In addition, the leaves of two-month-old *SlCER1–1*-knockdown tomato and one-month-old *SlCER1–1*-overexpressing *Arabidopsis* were also analyzed. Three independent biological replicates were tested for each experiment.

Total RNA was extracted using the OminiPlant RNA Kit (CWBIO), and 1 μg RNA was treated with DNase I (CWBIO) and reverse transcribed using the HiFiScript cDNA Synthesis Kit (CWBIO). The cDNA was diluted to 100 μl with ddH_2_O, and 2 μl of the template was used for qRT-PCR with the SYBR Green I Mix (ToYoBo, Osaka, Japan) in a total volume of 25 μl. The reaction was performed on an ABI StepOnePlus PCR system (Applied Biosystems) according to the manufacturer’s instructions. The tomato *SlActin4* gene (Solyc04g011500) and the *Arabidopsis AtACT8* gene (At1g49240) were selected as the respective internal controls. All primers are listed in Table S9. The 2^−ΔΔCt^ method was used to analyze relative gene expression levels [52].

### Generation of constructs

The cDNA from 80-day-old tomato leaves was amplified using primers specific for *SlCER1–1* and cloned into the XcmI site of the 35S promoter-driven pCXSN vector [53]. For the RNAi construct, a 553-bp specific fragment from the CDS of *SlCER1–1* was amplified and inserted forward and reverse into the multiple cloning sites of the pUCCRNAi intermediate vector [54]. The RNAi fragment was then amplified and cloned into the pCXSN vector as described above. The pro35S:SlCER1–1/eGFP construct was generated by amplifying and sub-cloning the *SlCER1–1* CDS without the termination codon into the modified pCAMBIA 2300 vector (kindly provided by Dr. Bing Jing). All primers are listed in Table S9.

### Subcellular localization and gene transformation

The endoplasmic reticulum (ER) marker mCherry-HDEL and the fusion construct pro35S:SlCER1–1/eGFP were co-transformed into the epidermal cells of *Nicotiana benthamiana* as previously described [55]. The cells were incubated for 2 days in the dark at room temperature and observed under a confocal microscope (Leica TCS SP4, Germany).

The *SlCER1–1* overexpression construct was transformed into wild-type *Arabidopsis* (Col-0) plants as previously reported [56]. Thirty T1 independent transgenic lines were confirmed by PCR, and their seeds were plated on half-strength MS medium containing 50 mg/l hygromycin to detect the 3:1 segregation ratio; 11 of the 30 independent transgenic lines were further screened to identify homozygous transgenic lines. Finally, five homozygous transgenic lines with varying levels of *SlCER1–1* expression were selected (Fig. S4). The *SlCER1–1* RNAi construct was transformed into the cotyledons of wild-type tomato (cv. Micro Tom) as previously reported [57]. More than 50 positive transgenic lines were confirmed by PCR and further screened by analyzing leaf wax contents using GC (data not shown). Three independent transgenic lines (lines 2, 5, and 19) with lower wax contents were selected.

### Cuticular wax analysis

To determine the wax developmental patterns on tomato leaves and fruits, all leaves of the fourth and fifth branches were sampled at 30, 60, 80, 100, and 120 days after germination. Immature green, mature green, breaker, orange, and red fruits were harvested as described by Mintz-Oron et al. [36]. To track cuticular wax accumulation under drought stress, one-month-old wild-type and *SlCER1–1* RNAi tomato plants were deprived of water for 13 days, and all leaves of the fourth and fifth branches were collected. Plants in the control group were watered as needed. To determine the effect of *SlCER1–1* on wax composition, the leaves of 60-day-old wild-type and *SlCER1–1* RNAi tomato plants, as well as their ripe (red stage) fruits, 40-day-old stems, and fully opened flowers without calyxes, were harvested. The rosette leaves of 1-month-old wild-type and *SlCER1–1 Arabidopsis* plants were also sampled. Three or four independent biological replicates were used for each experiment.

All materials were separately immersed in chloroform for 1 min, and then 20 μg n-tetracosane was added as an internal standard, and the wax mixtures were transferred to GC vials and dried under nitrogen gas. The extracted waxes were derivatized in 40 μl of pyridine and 40 μl of bis-(N, N-trimethylsilyl)-trifluoroacetamide (BSTFA) at 70°C for 60 min, and then the solution was dried again under nitrogen. Finally, each dried sample was dissolved in 500 μl chloroform for GC–MS (GCMS-QP2010, Shimadzu) and GC-FID (GC-2010 Plus, Shimadzu) analyses. The temperature was initially held at 50°C for 2 min. Then it was increased to 200°C at a rate of 20°C·min^−1^ and held for 1 min. Finally, it was further increased to 310°C at a rate of 1.6°C·min^−1^ and held for 20 min. The wax compounds were identified by comparison of their mass spectra with those of authentic standards and literature data, and they were quantified by comparison of FID peak areas with that of the internal standard. The surface areas of tomato leaves and fruits were calculated using ImageJ software, and the amount of wax was expressed per unit area. Fresh tomato flowers, stems, and *Arabidopsis* leaves were weighed on a microbalance, and their wax contents were expressed per gram fresh weight.

### Epidermal permeability analysis and RWC measurement

Leaf surface permeability was determined by chlorophyll leaching and water loss assays as described by Kosma et al. [47] with minor modifications. One-month-old wild-type and *SlCER1–1* RNAi tomato plants were kept in the dark for 3 hours, and their leaves were collected. For the chlorophyll leaching assay, the leaves were immersed in 50 ml 80% (v/v) ethanol. The amount of leached chlorophyll was measured every hour for 12 h at 647 nm and 664 nm using a U 60 spectrophotometer and expressed as the percentage of chlorophyll extracted at 24 h. For the water loss assay, the leaves were soaked in deionized water for 1 h in the dark, blotted with napkins to remove excess water, and weighed every hour for 12 h on a microbalance. The mature fruits were also harvested and placed in beakers at room temperature and then weighed every day for 21 days. The water loss rate was expressed as a percentage of the initial sample weight.

To measure leaf relative water content (RWC), one-month-old wild-type and *SlCER1–1* RNAi tomato plants were not watered for approximately 13 days, during which time most plants in the RNAi group withered. Plants in the control group were watered three times during this period. The leaves were immediately weighed on a microbalance after removal to obtain their fresh weights (FWs) and then immersed in ddH_2_O for 6 h. After excess water was blotted using napkins, the saturated fresh weights (TWs) were determined. The dry weights (DWs) of the leaves were recorded after drying in an oven at 65°C for 24 hours. Finally, RWC was calculated as (FW − DW) / (TW − DW) × 100. Three or four independent biological replicates were used for each experiment.

## Acknowledgments

This research was funded by the Program of Introducing Talents of Innovative Discipline to Universities (Project 111) from the State Administration of Foreign Experts Affairs (#B18042) and the Key Research and Development Project of Shaanxi Province (No. 2019ZDLNY04-05).

## Author Contributions

H.Q.W., C.L.L., and Z.H.W. planned and designed the research. H.Q.W., L.L., Y.F.C., T.X.L., Q.Q.J., and Z.Y.W. performed the experiments. H.Q.W. and L.L. analyzed the data. H.Q.W. and Z.H.W. wrote the manuscript, and C.L.L. revised it. All authors were involved in the revision of the manuscript and approved the final manuscript.

## Data Availability

All data pertaining to the present study have been included in the form of tables and/or figures in the present manuscript, and the authors are pleased to share analyzed/raw data and plant materials upon reasonable request.

## Conflict of interests statement

The authors declare no conflict of interest.

## Supplementary data

Supplementary data is available at *Horticulture Research Journal* online.

## Supplementary Material

Web_Material_uhac004
