# Supplementary material for: Tomato SlCER1–1 catalyzes the synthesis of wax alkanes, increasing drought tolerance and fruit storability
Source: Hortic Res. 2022 Feb 11;9:uhac004. doi: 10.1093/hr/uhac004 (PMC9071378; doi:10.1093/hr/uhac004)
Supplement: Web_Material_uhac004 [file web_material_uhac004.doc]

**Tomato SlCER1-1 catalyzes the synthesis of wax alkanes which increases the drought tolerance and fruit storability**

Hongqi Wu^1†^, Le Liu^1†^, Yaofeng Chen^1^, Tianxiang Liu^1^, Qinqin Jiang^1^, Zhengyang Wei^1^, Chunlian Li^1*^ and Zhonghua Wang^1*^.

**Table s1 Cuticular wax compositions and amounts in five developmental stages of tomato leaves**

| **Chemical class** | **Measurement** | **Five developmental stages of leaves** | | | | |
| --- | --- | --- | --- | --- | --- | --- |
|  |  | **30d** | **60d** | **80d** | **100d** | **120d** |
| **n-Alkanes** | **Amount (μg/cm^2^)** | 0.63 ± 0.06 | 2.16 ± 0.46 | 3.09 ± 0.37 | 6.06 ± 0.30 | 3.75 ± 0.93 |
|  | **Percentage (%)** | 38.03 ± 6.20 | 61.95 ± 6.71 | 67.63 ± 1.71 | 73.46 ± 2.31 | 71.27 ± 2.49 |
| **Branched alkanes** | **Amount (μg/cm^2^)** | 0.91 ± 0.27 | 1.14 ± 0.15 | 1.31 ± 0.22 | 1.83 ± 0.29 | 1.18 ± 0.43 |
|  | **Percentage (%)** | 53.71 ± 4.10 | 33.22 ± 7.14 | 28.48 ± 1.61 | 22.02 ± 1.65 | 21.94 ± 2.22 |
| **Triterpenoids** | **Amount (μg/cm^2^)** | 0.02 ± 0.01 | 0.08 ± 0.02 | 0.08 ± 0.02 | 0.20 ± 0.06 | 0.18 ± 0.07 |
|  | **Percentage (%)** | 1.25 ± 0.10 | 2.21 ± 0.21 | 1.80 ± 0.24 | 2.41 ± 0.50 | 3.43 ± 0.80 |
| **Primary alcohols** | **Amount (μg/cm^2^)** | 0.01 ± 0.00 | 0.00 ± 0.000 | 0.00 ± 0.00 | 0.00 ± 0.00 | 0.00 ± 0.00 |
|  | **Percentage (%)** | 0.59 ± 0.07 | 0.06 ± 0.01 | 0.05 ± 0.02 | 0.02 ± 0.00 | 0.03 ± 0.02 |
| **Aldehydes** | **Amount (μg/cm^2^)** | 0.01 ± 0.01 | 0.00 ± 0.00 | 0.00 ± 0.00 | 0.00 ± 0.00 | 0.00 ± 0.00 |
|  | **Percentage (%)** | 0.59 ± 0.17 | 0.06 ± 0.01 | 0.04 ± 0.03 | 0.04 ± 0.01 | 0.04 ± 0.01 |
| **Fatty acids** | **Amount (μg/cm^2^)** | 0.01 ± 0.01 | 0.00 ± 0.00 | 0.00 ± 0.00 | 0.00 ± 0.00 | 0.00 ± 0.00 |
|  | **Percentage (%)** | 0.72 ± 0.17 | 0.07 ± 0.02 | 0.05 ± 0.03 | 0.04 ± 0.00 | 0.06 ± 0.03 |
| **Unidentified** | **Amount (μg/cm^2^)** | 0.09 ± 0.06 | 0.09 ± 0.02 | 0.09 ± 0.01 | 0.17 ± 0.03 | 0.17 ± 0.05 |
|  | **Percentage (%)** | 5.10 ± 2.03 | 2.45 ± 0.41 | 1.94 ± 0.14 | 2.02 ± 0.29 | 3.22 ± 0.41 |
| **Total load** | **Amount (μg/cm^2^)** | 1.68 ± 0.38 | 3.47 ± 0.34 | 4.58 ± 0.60 | 8.26 ± 0.67 | 5.29 ± 1.46 |

Note. Amounts of individual wax constituents and total load are expressed as μg/cm^2^ leaf surface area, along with relative amounts of compound classes as % of total wax. Values indicate means of three biological replicates, and error bars indicate the standard deviations.

**Table s2 Cuticular wax compositions and amounts in five developmental stages of tomato fruits**

| **Chemical class** | **Measurement** | **Five developmental stages of fruits** | | | | |
| --- | --- | --- | --- | --- | --- | --- |
|  |  | **Immature green** | **Mature green** | **Breaker** | **Orange** | **Red** |
| **n-Alkanes** | **Amount (μg/cm^2^)** | 2.71 ± 0.41 | 3.12 ± 0.39 | 6.43 ± 1.12 | 6.22 ± 0.71 | 5.13 ± 0.39 |
|  | **Percentage (%)** | 47.92 ± 1.05 | 41.88 ± 1.91 | 62.09 ± 7.70 | 55.00 ± 1.21 | 56.92 ± 5.76 |
| **Branched alkanes** | **Amount (μg/cm^2^)** | 1.22 ± 0.29 | 0.92 ± 0.04 | 0.67 ± 0.15 | 1.64 ± 0.29 | 1.42 ± 0.83 |
|  | **Percentage (%)** | 21.54 ± 2.71 | 12.46 ± 1.02 | 6.42 ± 1.15 | 14.57 ± 2.87 | 15.37 ± 8.29 |
| **Triterpenoids** | **Amount (μg/cm^2^)** | 1.54 ± 0.11 | 2.98 ± 0.46 | 2.73 ± 0.57 | 2.97 ± 0.64 | 2.19 ± 1.02 |
|  | **Percentage (%)** | 27.59 ± 2.90 | 39.89 ± 2.03 | 26.59 ± 5.97 | 26.13 ± 3.04 | 23.49 ± 7.91 |
| **Primary alcohols** | **Amount (μg/cm^2^)** | 0.00 ± 0.00 | 0.00 ± 0.00 | 0.03 ± 0.01 | 0.04 ± 0.03 | 0.03 ± 0.03 |
|  | **Percentage (%)** | 0.04 ± 0.02 | 0.03 ± 0.01 | 0.31 ± 0.02 | 0.35 ± 0.16 | 0.38 ± 0.28 |
| **Aldehydes** | **Amount (μg/cm^2^)** | 0.00 ± 0.00 | 0.01 ± 0.00 | 0.00 ± 0.00 | 0.01 ± 0.00 | 0.01 ± 0.00 |
|  | **Percentage (%)** | 0.05 ± 0.01 | 0.06 ± 0.01 | 0.04 ± 0.00 | 0.06 ± 0.02 | 0.12 ± 0.02 |
| **Fatty acids** | **Amount (μg/cm^2^)** | 0.00 ± 0.00 | 0.00 ± 0.00 | 0.03 ± 0.01 | 0.03 ± 0.01 | 0.03 ± 0.02 |
|  | **Percentage (%)** | 0.06± 0.03 | 0.05 ± 0.02 | 0.25 ± 0.05 | 0.29 ± 0.05 | 0.36 ± 0.15 |
| **Unidentified** | **Amount (μg/cm^2^)** | 0.16 ± 0.04 | 0.42 ± 0.06 | 0.44 ± 0.06 | 0.41 ± 0.14 | 0.31 ± 0.17 |
|  | **Percentage (%)** | 2.81 ± 0.68 | 5.64 ± 0.42 | 4.30 ± 0.62 | 3.60 ± 0.83 | 3.36 ± 1.43 |
| **Total load** | **Amount (μg/cm^2^)** | 5.64 ± 0.73 | 7.44 ± 0.89 | 10.33 ± 1.11 | 11.32 ± 1.51 | 9.13 ± 1.54 |

Note. Amounts of individual wax constituents and total load are expressed as μg/cm^2^ fruit surface area, along with relative amounts of compound classes as % of total wax. Values indicate means of three biological replicates, and error bars indicate the standard deviations.

**Table s3 Cuticular wax composition of rosette leaves of different Arabidopsis lines**

| **Plant** | **Cuticular Wax Composition of Rosette Leaves** | | | | | | | |
| --- | --- | --- | --- | --- | --- | --- | --- | --- |
|  | **Total load** | **n-Alkanes** | **Branched alkanes** | **Primary alcohols** | **Branched alcohols** | **Aldehydes** | **Fatty acids** | **Unidentified** |
| **WT** | 31.35 ± 4.17 | 9.50 ± 1.87 | 0.46 ± 0.04 | 6.97 ± 1.06 | 0.76 ± 0.15 | 0.70 ± 0.06 | 1.87 ± 0.32 | 11.08 ± 0.85 |
| **#1** | 37.66 ± 3.41 | 17.01 ± 4.14 | 0.57 ± 0.28 | 7.79 ± 1.19 | 0.66 ± 0.02 | 0.68 ± 0.05 | 2.57 ± 0.84 | 8.38 ± 0.59 |
| **#2** | 46.64 ± 7.48 | 24.63 ± 7.18 | 0.71 ± 0.15 | 6.01 ± 0.84 | 0.54 ± 0.27 | 0.60 ± 0.07 | 1.93 ± 0.23 | 12.22 ± 2.35 |
| **#22** | 52.00 ± 2.65 | 27.02 ± 0.88 | 1.33 ± 0.22 | 6.97 ± 0.53 | 0.75 ± 0.31 | 0.84 ± 0.12 | 2.07 ± 0.34 | 13.03 ± 1.68 |

Note. Amounts of individual wax constituents and total load are expressed as μg/g leaf fresh weight. Values indicate means of three or four biological replicates, and error bars indicate the standard deviations.

**Table s4 Cuticular wax composition of leaves of different tomato lines**

| **Plant** | **Cuticular Wax Composition of Tomato Leaves** | | | | | | | |
| --- | --- | --- | --- | --- | --- | --- | --- | --- |
|  | **Total load** | **n-Alkanes** | **Branched alkanes** | **Triterpenoids** | **Primary alcohols** | **Aldehydes** | **Fatty acids** | **Unidentified** |
| **WT** | 3.18 ± 0.40 | 2.26 ± 0.29 | 0.34 ± 0.04 | 0.19 ± 0.04 | 0.05 ± 0.01 | 0.02 ± 0.00 | 0.07 ± 0.01 | 0.26 ± 0.03 |
| **#5** | 0.91 ± 0.10 | 0.24 ± 0.05 | 0.15 ± 0.03 | 0.13 ± 0.02 | 0.06 ± 0.01 | 0.04 ± 0.01 | 0.03 ± 0.01 | 0.26 ± 0.03 |
| **#19** | 0.82 ± 0.11 | 0.20 ± 0.06 | 0.13 ± 0.03 | 0.12 ± 0.02 | 0.05 ± 0.01 | 0.05 ± 0.03 | 0.04 ± 0.02 | 0.24 ± 0.02 |

Note. Amounts of individual wax constituents and total load are expressed as μg/cm^2^ leaf surface area. Values indicate means of four biological replicates, and error bars indicate the standard deviations.

**Table s5 Cuticular wax composition of fruits of different tomato lines**

| **Plant** | **Cuticular Wax Composition of Tomato Fruits** | | | | | | | |
| --- | --- | --- | --- | --- | --- | --- | --- | --- |
|  | **Total load** | **n-Alkanes** | **Branched alkanes** | **Triterpenoids** | **Primary alcohols** | **Aldehydes** | **Fatty acids** | **Unidentified** |
| **WT** | 5.73 ± 0.96 | 3.34 ± 0.65 | 0.38 ± 0.20 | 0.83 ± 0.30 | 0.11 ± 0.04 | 0.10 ± 0.03 | 0.21 ± 0.02 | 0.76 ± 0.09 |
| **#5** | 2.93 ± 0.44 | 0.69 ± 0.16 | 0.17 ± 0.03 | 1.17 ± 0.38 | 0.15 ± 0.05 | 0.06 ± 0.02 | 0.16 ± 0.02 | 0.53 ± 0.08 |
| **#19** | 2.62 ± 0.40 | 0.94 ± 0.16 | 0.18 ± 0.02 | 0.73 ± 0.14 | 0.11 ± 0.02 | 0.07 ± 0.01 | 0.13 ± 0.04 | 0.46 ± 0.06 |

Note. Amounts of individual wax constituents and total load are expressed as μg/cm^2^ fruit surface area. Values indicate means of four biological replicates, and error bars indicate the standard deviations.

**Table s6 Cuticular wax composition of stems of different tomato lines**

| **Plant** | **Cuticular Wax Composition of Tomato Fruits** | | | | | | | |
| --- | --- | --- | --- | --- | --- | --- | --- | --- |
|  | **Total load** | **n-Alkanes** | **Branched alkanes** | **Triterpenoids** | **Primary alcohols** | **Aldehydes** | **Fatty acids** | **Unidentified** |
| **WT** | 7.75 ± 0.49 | 3.99 ± 0.16 | 1.87 ± 0.04 | 0.07 ± 0.02 | 0.07 ± 0.00 | 0.04 ± 0.01 | 0.27 ± 0.03 | 1.44 ± 0.45 |
| **#5** | 3.62 ± 0.29 | 0.88 ± 0.13 | 0.91 ± 0.09 | 0.08 ± 0.02 | 0.16 ± 0.02 | 0.04 ± 0.01 | 0.23 ± 0.02 | 1.32 ± 0.09 |
| **#19** | 3.59 ± 0.19 | 0.81 ± 0.03 | 0.91 ± 0.03 | 0.06 ± 0.01 | 0.19 ± 0.02 | 0.04 ± 0.01 | 0.27 ± 0.01 | 1.31 ± 0.21 |

Note. Amounts of individual wax constituents and total load are expressed as μg/g stem fresh weight. Values indicate means of four biological replicates, and error bars indicate the standard deviations.

**Table s7 Cuticular wax composition of flowers of different tomato lines**

| **Plant** | **Cuticular Wax Composition of Tomato Flowers** | | | | | |
| --- | --- | --- | --- | --- | --- | --- |
|  | **Total load** | **n-Alkanes** | **Branched alkanes** | **Fatty acids** | **Triterpenoids & sterols** | **Unidentified** |
| **WT** | 0.43 ± 0.05 | 0.17 ± 0.02 | 0.22 ± 0.02 | 0.01 ± 0.00 | 0.01 ± 0.00 | 0.02 ± 0.00 |
| **#5** | 0.27 ± 0.06 | 0.10 ± 0.02 | 0.14 ± 0.03 | 0.01 ± 0.00 | 0.01 ± 0.00 | 0.02 ± 0.00 |
| **#19** | 0.23 ± 0.04 | 0.08 ± 0.02 | 0.12 ± 0.03 | 0.01 ± 0.00 | 0.01 ± 0.00 | 0.02 ± 0.00 |

Note. Amounts of individual wax constituents and total load are expressed as mg/g flower fresh weight. Values indicate means of three biological replicates, and error bars indicate the standard deviations.

**Table s8 Cuticular wax composition of leaves of different tomato lines under normal and drought conditions**

| **Plant** | **Cuticular Wax Composition of Tomato leaves** | | | | | | | |
| --- | --- | --- | --- | --- | --- | --- | --- | --- |
|  | **Total load** | **n-Alkanes** | **Branched alkanes** | **Triterpenoids** | **Primary alcohols** | **Aldehydes** | **Fatty acids** | **Unidentified** |
| **WT Control** | 1.92 ± 0.14 | 1.13 ± 0.09 | 0.60 ± 0.05 | 0.08 ± 0.02 | 0.02 ± 0.00 | 0.01 ± 0.00 | 0.01 ± 0.00 | 0.08 ± 0.00 |
| **WT Drought** | 2.86 ± 0.20 | 1.92 ± 0.18 | 0.70 ± 0.03 | 0.10 ± 0.01 | 0.02 ± 0.01 | 0.02 ± 0.01 | 0.01 ± 0.00 | 0.09 ± 0.01 |
| **#5 Control** | 0.79 ± 0.06 | 0.24 ± 0.04 | 0.15 ± 0.02 | 0.14 ± 0.02 | 0.06 ± 0.01 | 0.04 ± 0.01 | 0.01 ± 0.00 | 0.15 ± 0.02 |
| **#5 Drought** | 0.84 ± 0.13 | 0.25 ± 0.06 | 0.16 ± 0.04 | 0.14 ± 0.04 | 0.06 ± 0.01 | 0.05 ± 0.01 | 0.01 ± 0.00 | 0.17 ± 0.02 |
| **#19 Control** | 0.75 ± 0.07 | 0.22 ± 0.05 | 0.31 ± 0.03 | 0.07 ± 0.01 | 0.02 ± 0.00 | 0.03 ± 0.01 | 0.01 ± 0.00 | 0.08 ± 0.01 |
| **#19 Drought** | 0.71 ± 0.05 | 0.18 ± 0.02 | 0.29 ± 0.02 | 0.09 ± 0.01 | 0.02 ± 0.00 | 0.04 ± 0.01 | 0.01 ± 0.00 | 0.09 ± 0.01 |

Note. Amounts of individual wax constituents and total load are expressed are expressed as μg/cm^2^ leaf surface area. Values indicate means of four biological replicates, and error bars indicate the standard deviations.

| **Table s9 Sequences of primers used in cloning and PCR reactions** | | |
| --- | --- | --- |
| Names of genes or primers | Forward (5’—3’) | Reverse (5’—3’) |
|  | Primers used for qRT-PCR analysis | |
| *SlCER1-1* | ACTTGAAAGGCAAGGGAAATCA | CCAAGTAACCATCATAGACCACAAA |
| *SlCER1-2* | TTGGAAGGCTGGAACGAGAA | TGGATTGATCCGTAGAAGTAGCC |
| *SlCER1-3* | CGGGATTCTGCATGCATTGG | AGGCGTTGTCAATGGGCTAA |
| *SlCER1-4* | AGCACACCAGCCATGATTTG | CTCGTGCTCGTTCCAACCT |
| *SlCER1-5* | GGCTTCACAGAGCTTTACACCA | TGCAAATGGATGAATCACGGATG |
| *SlCER3-1* | GGACAAATGAAAGAGCACCAGA | TGGAGAAAGGCACTGAGCTG |
| *SlCER3-2* | CCCAAAATCATGGGAAATGCAC | GGTGCATGCATTGCAGAAGT |
| *SlActin4* | CTTGTCTGTGACAATGGAACTG | ATACCCACCATCACACCAGTAT |
| *AtACT8* | CCGAGCAGCATGAAGATTAAG | CATACTCTGCCTTAGAGATCCACA |
|  | Primers used for cloning of specific fragment in *SlCER1-1* CDS | |
| SlCER1-1i | CCGctcgagCTATTATTGGCTTCACAGAG | CGCggatccCCACTTAGAGGTGTAGGGTT |
|  | Primers used for cloning of RNAi fragment in pUCCRNAi vector | |
| RNAi | CCTGCAGGCTCGAGCCA | CCTGCAGGGTCGACCCAC |
|  | Primers used for cloning of full-length *SlCER1-1* CDS | |
| SlCER1-1-pCXSN | TTAGCATGGCTTCAAAACC | CATCAATGCCAGATCACA |
|  | Primers used for subcellular localization | |
| SlCER1-1/eGFP | ggatccctatcgattctagaATGGCTTCAAAACCAGGGATTC | cctttgcccatggctctagaTGCCTTTGATTCAGAAGCAGAA |
|  | Primers used for checking hygromycin gene | |
| *Hyg* | ATGTTGGCGACCTCGTATT | CGTTATGTTTATCGGCACTTT |

Note. Red lower-case letters in the SlCER1-1i primers indicate the restriction sites of XholI and BamhI, respectively. Lower-case letters in the SlCER1-1/eGFP primers indicate the fragments of the modified pCAMBIA 2300 vector.

**
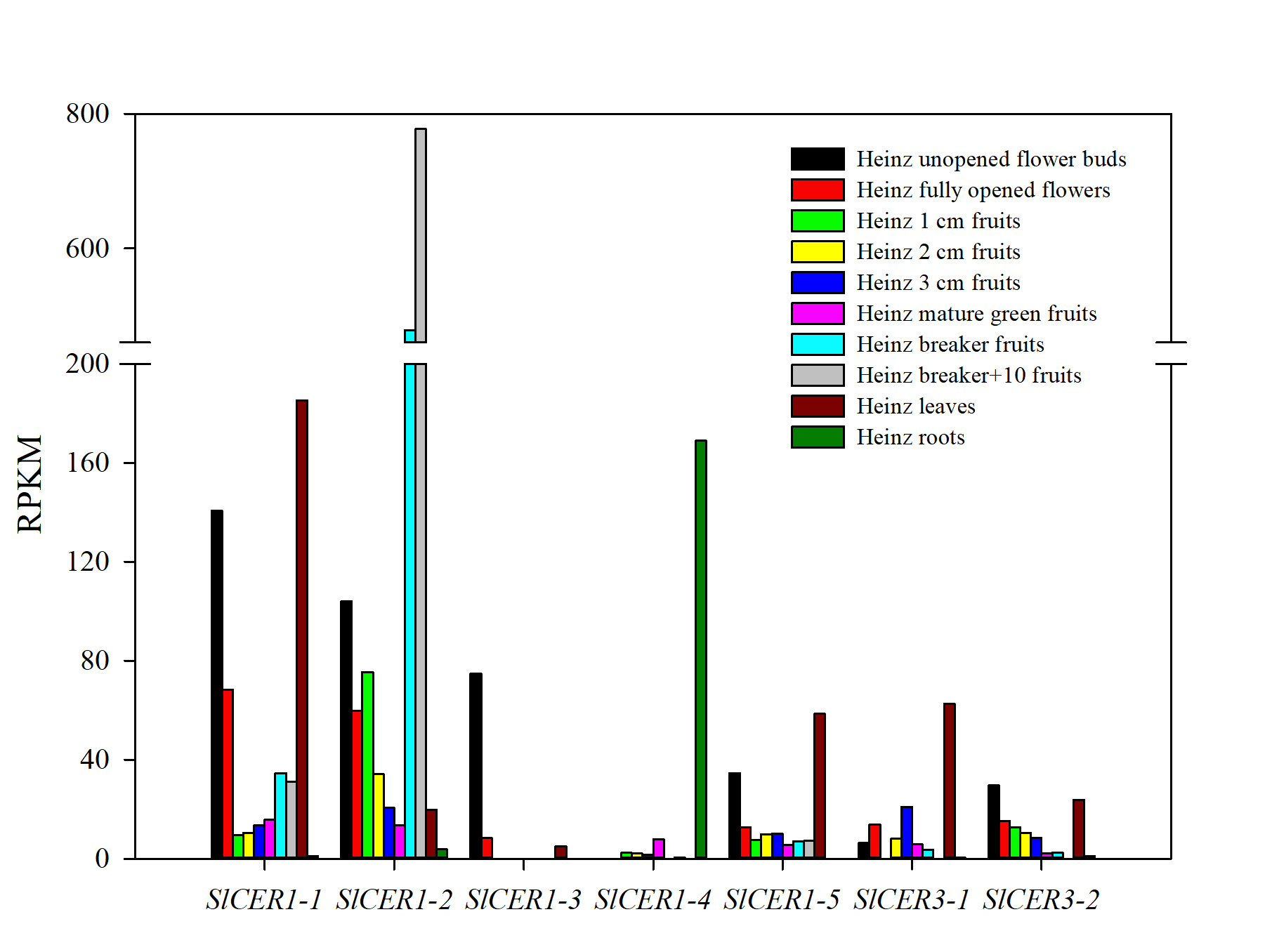
**

Fig.S1 Transcriptome analysis of *SlCER1* and *SlCER3* genes in various tissues. All samples come from tomato cultivar Heinz.


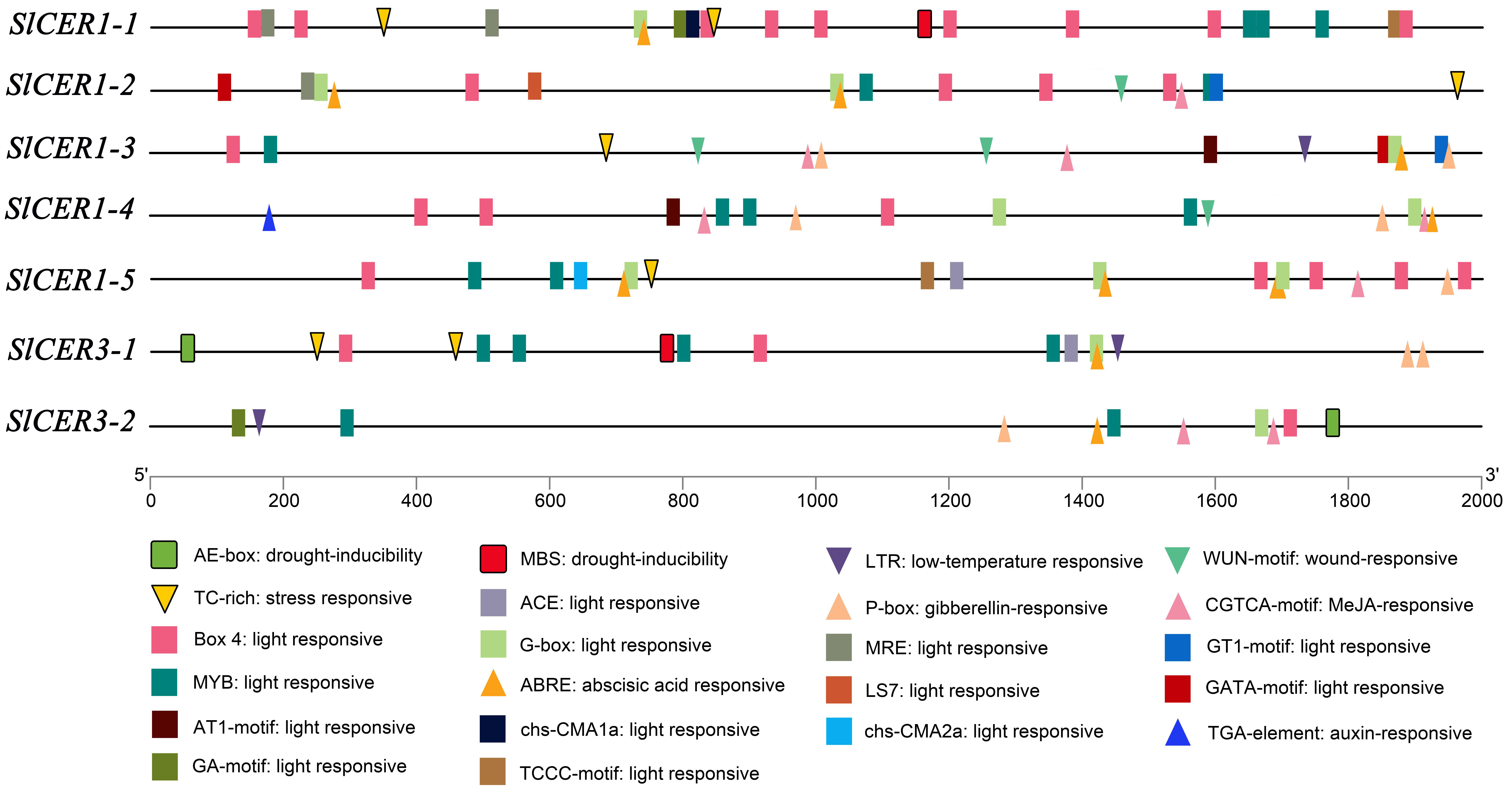


**Fig.S2 Promoter elements of *SlCER* genes predicted by PlantCARE**


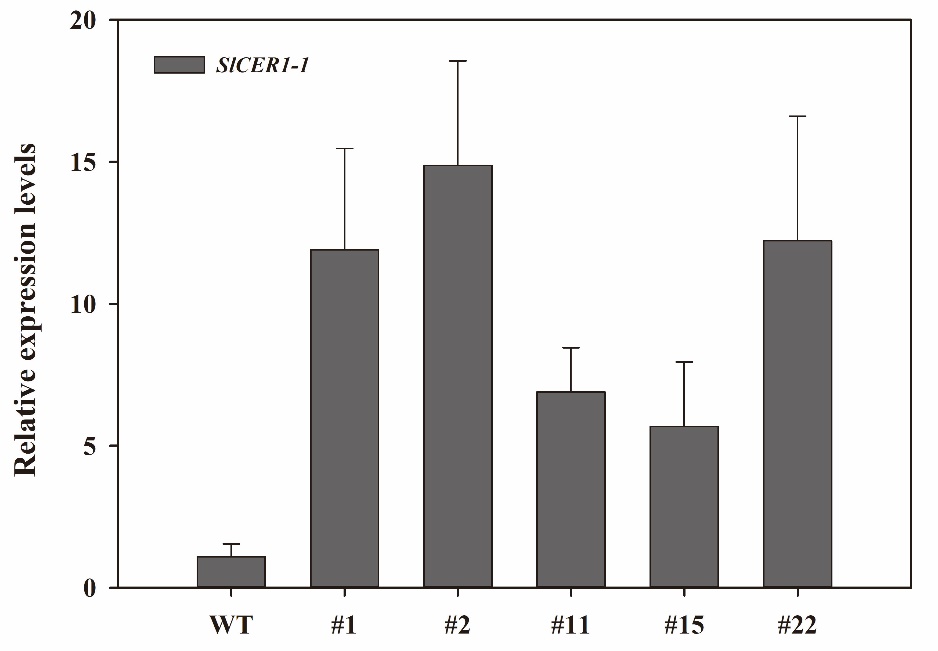


**Fig.S3 Expression analysis of *SlCER1-1* in five independent transgenic lines of Arabidopsis. Each value shows averages of three biological replicates, and error bars indicate the standard deviations.**


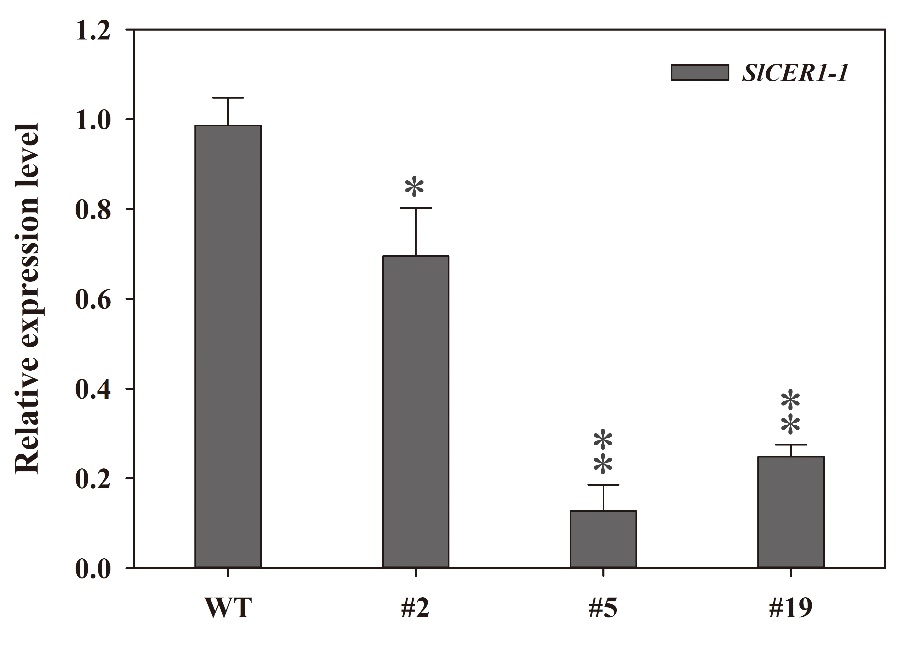


**Fig.S4 Expression analysis of *SlCER1-1* in tomato RNAi line 2, line5 and 19. Each value shows averages of three biological replicates, and error bars indicate the standard deviations. Asterisks point to significant differences from the WT plant (t-test: * for p < 0.05; ** for p < 0.01).**


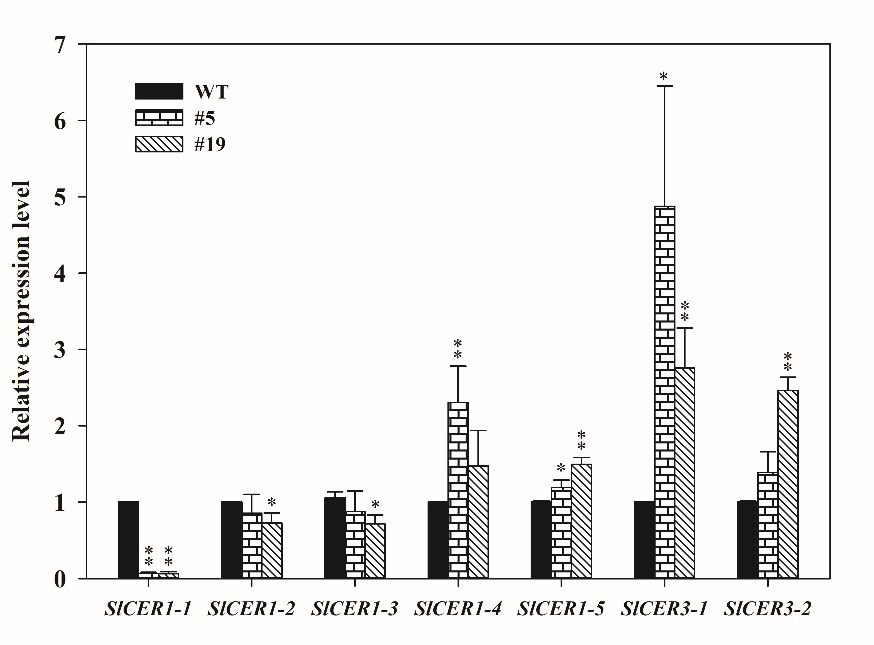


**Fig.S5 Expression analysis of *SlCER1s* and *SlCER3s* in tomato RNAi line 5 and 19.** Each value shows averages of three biological replicates, and error bars indicate the standard deviations. Asterisks point to significant differences from the WT plant (t-test: * for p < 0.05; ** for p < 0.01).
